# Draft Genome Sequence of *Lysinibacillus fusiformis* Strain Juneja, a Laboratory-Derived Pathogen of *Drosophila melanogaster*

**DOI:** 10.1128/genomeA.01571-17

**Published:** 2018-02-01

**Authors:** Brittny R. Smith, Robert L. Unckless

**Affiliations:** a Department of Molecular Biosciences, University of Kansas, Lawrence, Kansas, USA

## Abstract

*Drosophila melanogaster* is a model for the study of innate immunity, yet we have limited knowledge of its natural pathogens. In this study, we sequenced the genome of *Lysinibacillus fusiformis* strain Juneja, isolated from laboratory fly stocks. As a Gram-positive bacterium with unique peptidoglycans, this strain may provide a new model for pathogen recognition.

## GENOME ANNOUNCEMENT

We isolated a *Bacillus*-like strain from a laboratory stock of *Drosophila melanogaster*. When pricked with a needle dipped in a suspension of this strain (optical density at 600 nm [OD_600_], 3.977), adult males from *D. melanogaster* strain Canton-S showed a significantly increased mortality rate (76% survival 7 days postinfection compared to 100% survival after prick with a sterile needle [*P* < 0.001, Fisher exact test; more than 50 flies per each treatment]). This increased mortality rate is likely due to proliferation of the bacteria, since we also observed an approximately 20-fold increase in bacterial load in flies over a 24-h period, as assayed by spiral plating of fly homogenate.

*Bacillus* species are classified as Gram positive, but some members of the genus have a diaminopimelic acid-type (DAP-type) peptidoglycan, which is characteristic of Gram-negative bacteria (1). Members of the genus* Lysinibacillus*, however, produce a unique lysine-aspartate peptidoglycan (2). Therefore, a better understanding of the pathogen’s genome will inform our understandings of both pathogenicity and host immune defense.

To initially characterize the bacteria, we performed PCR and Sanger sequencing using universal 16S primers (E334F and E1115R) (3). We searched for sequence similarity against the 16S rRNA sequence database using the MegaBLAST program (4). The isolate showed 100% identity (664 bases) to several *Lysinibacillus fusiformis* strains. We named the strain *L. fusiformis* strain Juneja (for Punita Juneja, who isolated it).

High-molecular-weight DNA was prepared using the blood and cell culture DNA midi kit (product number 13343; Qiagen, Hilden, Germany) according to the manufacturer’s protocol for bacterial samples. Genomic DNA libraries were constructed using the Nextera DNA library prep kit (product number FC-121-1031; Illumina, Inc., San Diego, CA, USA), modified to conserve reagents (5). This library was then sequenced with an Illumina MiSeq system Nano run, yielding 761,837 paired-end 250-bp reads. We also used the MinION device (Oxford Nanopore Technologies, Oxford, UK) to obtain 3,251 long reads. Raw Illumina reads were filtered using Scythe (see https://github.com/vsbuffalo/scythe), and reads mapping to PhiX were discarded, yielding a total of 746,482 paired-end reads.

We assembled the *Lysinibacillus* genome using Unicycler (6) with default parameters and using both Illumina paired-end reads and MinION long reads. We assessed genome quality using QUAST (7), which revealed that the assembly consists of 22 contigs with an *N*_50_ of 818,181 bp and an *L*_50_ of 3. The GC content is 37.75%, and the total genome length is 4,546,138 bp. Both values are similar to those of the published genomes of *L. fusiformis* (37.3 to 37.6% GC content and 4,317,430- to 4,892,470-bp genome length).

The assembly also produced one complete plasmid genome of 32,702 bp, with a GC content of 39.5%. This plasmid is most similar to one found in *Lysinibacillus sphaericus* strain 2362 (92% identical over 97% of the plasmid). However, a plasmid found in *L. fusiformis* strain RB-21 also showed high similarity (90% identical over 99% of the plasmid). The genome and plasmid were annotated using the NCBI Prokaryotic Annotation Pipeline, which yielded 4,445 genes. Per NCBI policy, only contigs longer than 200 bp were included.

### Accession number(s).

The genome sequence of *Lysinibacillus fusiformis* strain Juneja has been deposited in GenBank under the accession number PDFK00000000.
