# Correction for Smith and Unckless, “Draft Genome Sequence of Lysinibacillus fusiformis Strain Juneja, a Laboratory-Derived Pathogen of Drosophila melanogaster”

**DOI:** 10.1128/genomeA.00475-18

**Published:** 2018-05-24

**Authors:** Brittny R. Smith, Robert L. Unckless

**Affiliations:** a Department of Molecular Biosciences, University of Kansas, Lawrence, Kansas, USA

## AUTHOR CORRECTION

Volume 6, no. 5, e01571-17, 2018, https://doi.org/10.1128/genomeA.01571-17. Page 2: The following statement should be added to Acknowledgments. “We thank the K-INBRE Bioinformatics Core (P20GM103418) for providing funds for the Oxford Nanopore Technologies MinION sequencer.”

